# 慢性淋巴细胞白血病中B细胞受体同型模式的生物学特征和临床意义

**DOI:** 10.3760/cma.j.cn121090-20230718-00012

**Published:** 2024-02

**Authors:** 彤璐 邱, 建勇 李, 奕 夏

**Affiliations:** 南京医科大学第一附属医院，江苏省人民医院血液科，南京医科大学血液研究重点实验室，江苏省肿瘤个体化医学协同创新中心，南京 210029 Department of Hematology, Key Laboratory of Nanjing Medical University, the First Affiliated Hospital of Nanjing Medical University, Jiangsu Province Hospital, Collaborative Innovation Center for Cancer Personalized Medicine, Nanjing 210029, China

## Abstract

慢性淋巴细胞白血病（CLL）是西方成人最常见的白血病，尽管CLL在亚洲人种中发病率偏低，但随着人口老龄化，CLL发病率在我国呈上升趋势。CLL细胞与微环境的交互中，B细胞受体免疫球蛋白（B-cell receptor immunoglobulin，BCR IG）对抗原的识别起重要作用，免疫球蛋白重链可变区（IG heavy variable region，IGHV）的突变状态更是判断CLL预后的经典标志物。超过40％的CLL患者间存在IGHV的偏向性使用和高度相似的重链可变区互补决定区3（heavy complementarity-determining region 3，HCDR3）氨基酸片段，此现象称为同型模式BCR。不同亚群的同型模式BCR存在独特的生物学和临床特征。其中IGHV有突变但预后差的#2亚群和发生Richter转化风险高的#8亚群被欧洲CLL研究启动组推荐纳入IGHV突变状态的临床报告之中。本文综述CLL中同型模式BCR的定义、分布、生物学特征和临床意义。

## Abstract (en)

Chronic lymphocytic leukemia (CLL) is the most common leukemia in the Western adults, although the incidence of CLL is relatively low in Asian populations. However, with the aging population, the incidence of CLL is increasing in China. The interaction between CLL cells and the microenvironment plays a crucial role in the recognition of antigens by the B-cell receptor immunoglobulin (BCR IG). The mutational status of the immunoglobulin heavy variable region (IGHV) is a classical prognostic marker for CLL. Over 40% of CLL patients exhibit biased usage of IGHV and highly similar amino acid sequences in the heavy complementarity-determining region 3 (HCDR3), known as the BCR stereotypy. Different subgroups of stereotyped BCR exhibit distinct biological and clinical features. Among them, subset #2 with mutated IGHV and poor prognosis, as well as the subset #8 with a high risk of Richter transformation, have been recommended by the European Research Initiative on CLL to be included in clinical reports on IGHV mutational status. This review summarizes the definition, distribution, biological characteristics, and clinical significance of clonality patterns of the BCR in CLL.

免疫球蛋白（IG）是B细胞受体（BCR）的组成部分。免疫球蛋白重链可变区（IG heavy variable region，IGHV）-多样性（diversity，D）-连接区（junction，J）重排被作为克隆性标志，广泛应用于B淋巴细胞肿瘤的诊断和疗效监测。慢性淋巴细胞白血病（CLL）是西方成人最常见的白血病，临床表现为大量CD5^+^CD23^+^的成熟单克隆B淋巴细胞积聚于外周血、骨髓及淋巴器官[1]。CLL病程异质性强，可根据IGHV突变状态分为不同的预后群体：IGHV有突变（与胚系V区基因序列的相似性<98％）的患者预后好，而IGHV无突变（与胚系V区基因序列的相似性≥98％）的患者预后差[2]–[3]。

与正常的CD5^+^ B淋巴细胞相比，CLL细胞存在IGHV基因限制性使用，更常使用IGHV1-69、IGHV4-34、IGHV4-39等基因片段[4]。在无亲属关系、间隔遥远的不同CLL患者之中发现了被称为同型模式BCR（BCR stereotypy）的高度近似的IG重链可变区互补决定区3（heavy complementarity-determining region 3，HCDR3）序列，提示特定的选择压力可能在CLL发病中起到重要作用[5]–[7]。Agathangelidis等[8]报道在以西方人种为主的29 856例CLL患者中，41％存在同型模式BCR的使用，且不同的同型模式BCR亚群（subset）患者具有不同的临床预后和生物学特征。2022年欧洲CLL研究启动组（ERIC）CLL IG序列分析指南中推荐，除了IGHV突变状态外，所有报告至少应包含同型模式BCR #2、#8亚群的检测[9]。同时，第5版世界卫生组织（WHO）白血病淋巴瘤分类中也指出，对CLL的预后判断必须包含同型模式BCR，尤其是#2亚群的检测[10]。国内学界对同型模式BCR的认知尚不足，且相较于西方患者，我国CLL患者在同型模式BCR的使用特征上存在显著不同[11]，因此，本文拟就CLL中同型模式BCR的生物学特征和临床意义作综述。

一、同型模式BCR的概述

21世纪初，学者们发现大部分重链使用IGHV3-21基因的CLL患者轻链都使用IGLV3-21基因，且具有高度相似的HCDR3区氨基酸序列[12]–[13]。正常B细胞由于不同V-D-J基因重排导致的组合多样性和重排过程中V-D-J基因片段之间连接的多样性，两个不同克隆的B淋巴细胞使用相同HCDR3区的可能性<10^−12^。因此，CLL中这种不同患者间具有高度类似的HCDR3区氨基酸序列的现象并非偶然，而是由疾病发生过程中的选择压力所致[7]。当≥2例患者的肿瘤细胞使用相同或近似的HCDR3区氨基酸序列，即可被认为存在同型模式BCR。其中仅由2例患者组成的群体称为亚组（cluster），而≥3例患者组成的群体称为亚群（subset）。

鉴定同型模式BCR采用的是Agathangelidis等[6]于2012年提出的标准：①HCDR3氨基酸长度相同；②HCDR3同一位置氨基酸相同性≥50％；③HCDR3同一位置氨基酸相似性≥70％［根据国际免疫遗传学数据库（International ImMunoGeneTics information system，IMGT），相似氨基酸为AVLI、CM、ST、KRH、DE、NQ、F、W、P、G、Y］；④HCDR3区采用亚群特定的氨基酸排列基序（motifs）；⑤IGHV家族属于同一集团（clan，IGHV1/5/7家族为clan 1，IGHV2/4/6家族为clan 2，IGHV3家族为clan 3）。与已有序列相比，患者序列必须同时满足以上5条标准，方可被认为属于该同型模式BCR亚群或亚组。

Agathangelidis等[8]在2021年更新的包含29 856例CLL患者的30 413个IGHV-D-J序列的报道中，已发现2 000余个CLL同型模式BCR亚群及亚组，其中单个亚群序列数占总序列数的比例≥0.2％的主要亚群（major subset）共有29个，其余占总序列数的比例<0.2％的亚群/亚组被归类为次要亚群（minor subset）。在西方CLL患者中，最常见的主要亚群依次为：#1、#2、#4、#5及#6亚群。表1概括了29个主要亚群的使用率、结构和基因使用片段。值得注意的是其中部分亚群的患者具有较为相似的基因突变及分子遗传学特征，且该现象并不少见。除下文提到的#1，#2，#4及#8亚群外，#5亚群患者中常见del（11q），#6亚群患者中NOTCH1突变发生率高、del（17p）常见，常见于年轻患者的#77亚群中del（13q）发生率高[8]。

**表1:** 同型模式BCR主要亚群的结构和临床特征

亚群#	IGHV突变状态	比例（%）	IGHV使用基因片段	IGHD使用基因片段	IGHJ基因	HCDR3长度	HCDR3基序
1	多数无突变	2.15	Clan I	IGHD6-19 ^a^	IGHJ4	13	ARxQWLxxxxFDY
2	多数突变	2.53	IGHV3-21	-	IGHJ6	9	Ax[DE]xxxMDV
4	突变	0.88	IGHV4-34	IGHD5-18 ^a^	IGHJ6	20	[AV]RGxxxxxxx[RK]RYYYYGMDV
5	无突变	0.50	IGHV1-69	IGHD3-10 ^a^	IGHJ6	20	ARxxxxGV[IV]xxxYYYY[GY]MDV
6	无突变	0.81	IGHV1-69	IGHD3-16	IGHJ3	21	ARGGxYDY[VI]WGSYRxNDAFDI
8	无突变	0.50	IGHV4-39	IGHD6-13	IGHJ5	19	A[RST]xxxYSSSWYxxxNWFDP
8B	无突变	0.22	IGHV4-39	IGHD6-13	IGHJ5	18	A[RST]xxxYSSSWYxxxWFDP
10	无突变	0.29	IGHV4-39	IGHD2-2	IGHJ6	22	AR[HD]RxGYCSSTSCYYYYYGMDV
12	无突变	0.24	IGHV1-2, IGHV1-46	IGHD3-22	IGHJ4	19	ARDxxYYDSSGYY[ST]xxFDY
14	突变	0.23	IGHV4-4^a^	-	IGHJ4	10	[AV]RGGxWxFDx
16	突变	0.27	IGHV4-34	IGHD2-15, IGHD2-2	IGHJ6	24	AxRFYCSGxxCxxxxYYYYYG[LM]D[VA]
31	无突变	0.26	IGHV3-48	IGHD3-3	IGHJ6	21	AR[DE]xDFWSGYYxYYYYY[GY]MDV
59	无突变	0.34	IGHV1-58, IGHV1-69	IGHD3-3	-	12	AxxxDFWSGYxx
64B	无突变	0.26	IGHV3-48 ^a^	-	IGHJ6	21	A[KR][DE][ST][PL]LVV[PV][AT]AI[FY]YYYYGMDV
73	突变	0.21	IGHV3-30 ^a^	IGHD6-13^a^	IGHJ4	12	A[KR]Dxxxx[WY]xxDY
77	突变	0.43	IGHV4-4, IGHV4-59	IGHD6-19 ^a^	IGHJ4	14	[AV]RG[PA][DN]x[ST]GWxx[FL]xY
99	大多数无突变	0.40	Clan I	IGHD6-19 ^a^	IGHJ4	14	ARxQWLxxxxxFDY
111	大多数突变	0.20	IGHV1-18	IGHD2-15 ^a^	-	10	ARxSGGxx[DE]x
169	大多数无突变	0.21	IGHV3-48	IGHD1-26 ^a^	-	9	AR[DE]xxxxxx
188	大多数突变	0.23	IGHV3-72	IGHD2-2	-	17	[AV]RxxYC[ST][SG][TG][TS]CRxx[FL]Dx
201	突变	0.23	IGHV4-34	-	IGHJ3	17	ARRxxxWxxxxxD[AG]FDx
252	突变	0.22	IGHV3-30	IGHD3-10 ^a^	IGHJ6	19	AK[VI]xxxGxFxxxxYYGMD[VA]
148B	突变	0.50	IGHV2-5	-	IGHJ4	17	AHRxxxxxxWxxGxFDY
28A	无突变	0.20	IGHV1-2	IGHD1-26	IGHJ6	17	ARx[YL]SGSYYYYYYGMDV
3C2	无突变	0.22	IGHV1-69	IGHD2-2	IGHJ6	22	AxxxDIVVVPAAxxYYYYGMDV
3C3	无突变	0.32	IGHV1-69	IGHD2-2	IGHJ6	22	ARxxPDIVVVPAAIx[YR]YYGMDV
7C2	无突变	0.24	IGHV1-69	IGHD3-3	IGHJ6	23	A[RST]xxxxDFWSGYYPNYYYYGMDV
7D3	无突变	0.22	IGHV1-69	IGHD3-3	IGHJ6	24	Axxxxx[YGD]DFWSGYYPNYYYY[GY]MDV
202	无突变	0.23	Clan III	IGHD4-17 ^a^	-	14	ARGxxGDYxxxFD[YIV]

注 BCR：B细胞受体；IGHV：免疫球蛋白重链可变区；IGHD：免疫球蛋白重链多样性；IGHJ：免疫球蛋白重链连接区；HCDR3：IG重链可变区互补决定区3；a：大多数；-：基因片段/基因种类较多

除了主要亚群和次要亚群的分类之外，报道中还提出了新的“卫星亚群”（satellite subset）的概念[8],[14]。卫星亚群的定义为：①与对应的主要亚群使用同一集团的IGHV基因；②HCDR3长度差异不超过2个氨基酸；③使用主要亚群特定的HCDR3氨基酸排列基序。29个主要亚群中，存在5对卫星亚群：#1和#99亚群、#2和#169亚群、#3C2和#3C3亚群、#7C2和#7D3亚群以及#8和#8B亚群。这些卫星亚群与相对应的主要亚群的免疫遗传学和临床特征高度相似，如#7C2与#7D3亚群患者中均常见del（11q）和del（17p），#3C2及#3C3亚群患者中SF3B1突变率高，del（11q）常见。此外还有更多的次要亚群属于卫星亚群，例如#2亚群除#169外还有6个隶属于次要亚群的卫星亚群，这8个群体相加，使#2亚群样的患者比例从原先的2.5％增加到了2.9％。

二、同型模式BCR亚群的遗传学特征和临床意义

同型模式BCR亚群众多，各个亚群具有截然不同的特征（表1）。但由于样本量小，绝大多数同型模式BCR亚群的临床意义并不明确。本文介绍研究深入、临床意义明确的主要亚群#1、#2、#4及#8。

1. #1亚群：同型模式BCR#1亚群的重链由IGHV集团1（即IGHV1/5/7家族）基因和IGHD6-19、IGHJ4基因组成，轻链由IGKV1（D）-39和IGKJ1-2组成。#1亚群的使用者IGHV无突变， NOTCH1、TP53、NFKBIE基因突变和del（11q）常见，发生率分别为27％、16％、15％和22％[15]–[16]。#1亚群患者预后较差，中位至首次治疗时间（TTFT）为1.6年，采用免疫化学治疗中位总生存（OS）期为8.3年，短于其他IGHV无突变但不属于同型模式BCR的CLL患者[17]–[18]。与非#1亚群患者相比，#1亚群患者具有独特的基因表达谱（GEP）特征，差异表达基因涉及BCR、细胞凋亡、氧化磷酸化、肌动蛋白细胞骨架调控、趋化因子等多种信号通路[19]。此外，#1亚群CLL细胞EZH2表达水平显著升高[20]。在原代CLL细胞的体外实验中，EZH2的表达水平与CpG诱导的Toll样受体9（TLR9）激活呈正相关，联合使用EZH2抑制剂与BCR信号通路抑制剂伊布替尼可协同杀伤CLL细胞[21]。

2. #2亚群：同型模式BCR #2亚群以重链IGHV3-21基因和轻链IGLV3-21*01基因的使用为特征。在西方CLL患者群体中，#2亚群是占比最高的亚群，使用率约为2.5％。#2亚群的CLL患者SF3B1基因突变、del（11q）、del（13q）更常见，发生率分别为45％、25％和50％，但TP53基因异常的发生率仅为2％。尽管约60％使用#2亚群的CLL患者IGHV有突变，但预后仍差，中位TTFT为1.9年，采用免疫化学治疗中位OS期为10.7年，与既往采用单纯化学治疗的数据相比无明显改善[15],[17]–[18]。#2亚群CLL细胞存在独特的轻链介导的细胞自主性（cell-autonomous）BCR活化信号：IGLV3-21*01铰链区上的点突变R110（G变为R）导致相邻BCR同源二聚体化，为CLL细胞提供了持续的自身活化信号。#2亚群BCR IG的自身识别亲和力低且解离快，在受体交联后#2亚群CLL细胞持续存在高反应性的BCR，与其侵袭性的病程相吻合[22]–[23]。同样使用IGLV3-21*01的卫星亚群#169等亦存在IGLV3-21*01 R110突变，因此也存在肿瘤细胞自主性活化的现象[14]。尽管此类患者采用化学免疫治疗预后差，但小样本数据显示，以BTK抑制剂、BCL2抑制剂为主的靶向治疗可以改善IGLV3-21*01 R110突变患者的预后[24]。

3. #4亚群：同型模式BCR #4亚群由IGHV4-34、IGHD3-10和IGHJ6组成，IGHV有突变。#4亚群的CLL患者年轻，预后好，TTFT长达11年，55％存在del（13q）[15],[17]。与IGHV无突变的#1、#8等亚群不同，在以#4亚群为代表的IGHV有突变的同型模式BCR亚群中HCDR3区的路标性（landmark）氨基酸少，却在整个IGHV编码区域具有高度类似的点突变特征，因此形成亚群特异性的体细胞高频突变（somatic hypermutation，SHM）模式。BCR IG晶体学分析显示，#4亚群的IgG受体内的结合位点可通过HCDR3介导的与BCR内部的构象表位的相互作用实现特异性、长期、高亲和力的自身识别，激活细胞内信号级联，形成CLL细胞自主性的内源性信号传导。与#2亚群不同的是，#4亚群的BCR IG自身识别亲和力高且持久，亚群特异的SHM模式稳定了受体结构，增强了信号传导复合物的半衰期，导致了B细胞无能（anergy）和惰性病程[23]。然而，并非所有使用IGHV4-34的同型模式BCR亚群都具备此特征，使用IGHV4-34的另一个主要亚群，#16亚群，就与#4亚群有截然不同的SHM模式和GEP特征，提示即使在使用相同IGHV基因的CLL群体中，仍存在不同的选择压力和发病机制[25]。

4. #8亚群：同型模式BCR #8亚群由重链IGHV4-39-IGHD6-19-IGHJ5组成。使用#8亚群的患者IGHV无突变，NOTCH1基因突变、+12和del（17p）常见，发生率分别为30％、60％和15％[15],[17]。#8亚群患者预后差，中位TTFT为1.5年，采用免疫化学治疗中位无进展生存（PFS）期为26.3个月，中位OS期为4～7年[11],[16]–[17]。Rossi等[26]发现#8亚群患者发生Richter转化的风险高，5年转化率高达68.7％。另一项来自德国CLL研究组的meta分析整合了CLL8、CLL10和CLL11等3项前瞻性临床研究数据，发现晚期患者中6.3％的#8亚群患者发生了Richter转化，显著高于其他非#8亚群的IGHV无突变患者[16]。因此，ERIC推荐对#8亚群的CLL患者进行更加严密的临床监测，疾病进展时视情况加做PET-CT检查从而及时识别可能发生的Richter转化[9]。与病程侵袭的#1和#2亚群CLL相比，#8亚群CLL细胞产生的重组抗体具有更强烈的多抗原反应性，可以和包括Sm、Jo-1、U1-RNP、SSA/Ro在内的自身抗体，以及细菌、病毒等微生物抗原表位，细胞凋亡过程中产生的新表位，甚至正常人类上皮细胞发生反应；并且#8亚群CLL细胞被上述抗原激活后，可以产生快速而显著的BCR和TLR信号通路激活，这种持续不断的抗原刺激信号可能有利于#8亚群CLL细胞的扩增和生存，为其高危突变的积累和组织学转化提供先决条件[27]。另一方面，#8亚群具有独特的表观遗传学特征。Papakonstantinou等[28]通过全基因组DNA甲基化芯片发现，相比于#6亚群，#8亚群CLL患者的差异甲基化CpG岛（differentially methylated CpG sites，DMCpGs）更多地呈现低甲基化状态，且低甲基化的CpG岛多位于启动子区域。结合RNA测序数据，TP63基因同时存在启动子区低甲基化和mRNA高表达，被认为是导致#8亚群生物学高侵袭性的原因之一。近期Tsagiopoulou等[29]对46例CLL患者的肿瘤细胞进行了全基因组染色质H3K27ac乙酰化图谱检测，也发现与#1、#2、#4亚群和其他非同型模式BCR的CLL患者相比，#8亚群CLL呈现全然不同的染色质活化图谱，却与非#8亚群的Richter转化患者高度相似。

综上，不同的同型模式BCR亚群具有独特的IG结构，并与临床及生物学特征紧密关联：#1、#2、#8亚群患者预后差，#4亚群患者预后好；TP53、NOTCH1基因异常常见于#1、#8亚群，SF3B1基因突变常见于#2亚群；#8亚群具有不同于其他亚群的表观遗传学和抗体反应性特征。然而，导致这些关联的原因和同型模式BCR的成因仍有待探索。

三、同型模式BCR的地域差异

既往研究表明，IGHV突变状态、同型模式BCR使用率及常见亚群的分布存在着地域差异。与西方CLL患者相比，中国患者存在以下IG特征：①IGHV有突变的患者比例更高（62.6％ 对54.1％）；②IGHV基因片段的使用率不同：中国患者使用IGHV4-34（11.8％ 对8.9％）、IGHV4-39（11.8％对8.9％）、IGHV4-59（3.9％对 2.8％）的比例更高，而使用IGHV1-69（6.1％对12.8％）、IGHV1-2（1.9％对4.6％）的比例更低；③同型模式BCR的分布不同：中国患者使用主要亚群的比例更低（8.4％对13.5％）、更偏向使用#8亚群（1.8％对0.5％）及其卫星亚群#8B（0.9％对0.2％），而西方患者中最常见的#2亚群在中国患者中罕见（0.2％对2.5％），常见于#2亚群使用者的SF3B1突变在中国CLL患者中也被发现突变率偏低[11],[30]–[31]。CLL患者IG基因的地域差异并非偶然，也不仅体现在中国与西方患者的比较中。与北欧CLL患者群相比，南欧患者IGHV4-59的使用率偏高，而IGHV3-21、IGHV6-1的使用率偏低[8]；俄罗斯的CLL患者IGHV无突变的比例达到68％，IGHV1家族使用率34.3％，IGHV1-69使用率21.3％，#1使用率3.3％，均高于其他地区的报道[32]；中国台湾地区CLL患者IGHV无突变的比例仅占20％~30％，最常见的同型模式BCR亚群方面，Huang等[33]报道为#8和#1亚群，Yao等[34]则报道为#77和#28A亚群。尽管均为回顾性研究，以上数据均证实地域差距对CLL的免疫遗传学特征造成影响（表2）。

**表2:** 不同地区慢性淋巴细胞白血病患者中IG基因使用片段及同型模式BCR亚群的差异

参考文献	民族	患者数量（IG序列数量）	IGHV有突变∶无突变（%）	最常见的IGHV基因	同型模式BCR分布（主要亚群/次要亚群，%）	最常见的同型模式BCR亚群
Biderman等[32]	高加索人（俄罗斯）	1 800（1 800个功能性重排）	32∶68	IGHV1-69, IGHV4-34, IGHV3-23	NA（13.4/NA）	#1, #6, #2
Agathangelidis等[14]	高加索人	7 424（7 596个功能性重排）	54.9∶45.1	IGHV1-69, IGHV4-34, IGHV3-23	30.4（12.4/18.0）	#2, #1, #4
Agathangelidis等[8]	高加索人为主	29 856（30 413个功能性重排）	54.1∶45.9	IGHV1-69, IGHV4-34, IGHV3-23	41.2（13.5/27.7）	#2, #1, #4
Marinelli等[11]	高加索人	789（792个功能性重排）	48.9∶51.1	IGHV1-69, IGHV4-34, IGHV3-23	25.7（14.5/11.2）	#1, #4, #2
	亚洲人	623（631个功能性重排）	65.7∶34.2	IGHV4-34, IGHV3-23, IGHV3-7	19.7（10.5/9.2）	#8, #1, #4
cwCLL	亚洲人（中国）	3 147（3 154个功能性重排）	62.6∶37.4	IGHV4-34, IGHV3-23, IGHV4-39	NA（8.4/NA）	#8, #1, #4
Yao等[34]	亚洲人（中国台湾）	255（243个功能性重排）	77.8∶22.2	IGHV3-7, IGHV4-34, IGHV3-23	34.2（6.2/28.0）	#77, #28A, #1
Huang等[33]	亚洲人（中国台湾）	194 （191个功能性重排）	71.2∶28.8	IGHV3-23, IGHV3-7, IGHV3-74	18.3（8.9/9.4）	#8, #1, #2

注 BCR：B细胞受体；IG：免疫球蛋白；IGHV：免疫球蛋白重链可变区；重链连接区；HCDR3：IG重链可变区互补决定区3；NA：不适用

四、总结与未来方向

同型模式BCR的使用是CLL普遍而独特的现象，少见于其他B淋巴细胞来源的肿瘤[5]。一方面，它提示抗原的选择压力在CLL发生发展过程的作用；另一方面，正如#2亚群往往IGHV有突变却对免疫化学治疗反应不佳、而#8亚群发生Richter转化比例高一样，不同同型模式BCR亚群的使用是对仅靠IGHV突变状态判断预后的重要补充。尽管目前大量数据提示BTK抑制剂可以克服IGHV无突变带来的不良预后影响，但是BTK抑制剂对不同同型模式BCR亚群的疗效还有待挖掘[35]–[37]。此外，国外也有研究者尝试利用#1亚群和#2亚群的同型模式BCR衍生肽段构建亚群特异性的CLL疫苗，在体外实验和小鼠模型中取得了初步成效[38]，提示这种特殊的、固定的BCR结构模式同样具有成为治疗靶点的潜力。综上，同型模式BCR亚群在CLL的基础和临床研究中均值得进一步探索，目前，由中国慢性淋巴细胞白血病工作组（Chinese Workshop on CLL，cwCLL）牵头的全国CLL患者IG基因和同型模式BCR使用的回顾性研究正在进行。
